# Computational Identification of Druggable Bioactive Compounds from *Catharanthus roseus* and *Avicennia marina* against Colorectal Cancer by Targeting Thymidylate Synthase

**DOI:** 10.3390/molecules27072089

**Published:** 2022-03-24

**Authors:** Md Rashedul Islam, Md Abdul Awal, Ahmed Khames, Mohammad A. S. Abourehab, Abdus Samad, Walid M. I. Hassan, Rahat Alam, Osman I. Osman, Suza Mohammad Nur, Mohammad Habibur Rahman Molla, Abdulrasheed O. Abdulrahman, Sultana Rajia, Foysal Ahammad, Md Nazmul Hasan, Ishtiaq Qadri, Bonglee Kim

**Affiliations:** 1 Department of Chemistry, Faculty of Science, King Abdul-Aziz University, Jeddah 21589, Saudi Arabia; rashed.pharm.jnu@gmail.com (M.R.I.); whassan@kau.edu.sa (W.M.I.H.); oabdelkarim@kau.edu.sa (O.I.O.); 2 Department of Pharmacy, Faculty of Life and Earth Sciences, Jagannath University, Dhaka 1100, Bangladesh; 3 Department of Pharmacy, Varendra University, Rajshahi 6204, Bangladesh; rajia_bio@yahoo.com; 4 Department of Biochemistry, Faculty of Science, King Abdul-Aziz University, Jeddah 21589, Saudi Arabia; pharmacist_awal@yahoo.com (M.A.A.); snur0001@stu.kau.edu.sa (S.M.N.); roabdulrahman@gmail.com (A.O.A.); 5 Department of Pharmaceutics and Industrial Pharmacy, College of Pharmacy, Taif University, P.O. Box 11099, Taif 21944, Saudi Arabia; dr.akhamies@gmail.com; 6 Department of Pharmaceutics, Faculty of Pharmacy, Umm Al-Qura University, Makkah 21955, Saudi Arabia; maabourehab@uqu.edu.sa; 7 Department of Pharmaceutics and Industrial Pharmacy, College of Pharmacy, Minia University, Minia 61519, Egypt; 8 Department of Genetic Engineering and Biotechnology, Jashore University of Science and Technology, Jashore 7408, Bangladesh; samad_gebt@just.edu.bd (A.S.); rahatalam1643@gmail.com (R.A.); 9 Laboratory of Computational Biology, Biological Solution Centre (BioSol Centre), Jashore 7408, Bangladesh; 10 Department of Biological Sciences, Faculty of Science, King Abdul-Aziz University, Jeddah 21589, Saudi Arabia; saky7009@gmail.com; 11 Institut Cochin, Université de Paris, Inserm, 75014 Paris, France; 12 Center for Interdisciplinary Research (CIR), Varendra University, Rajshahi 6204, Bangladesh; 13 Laboratory of Pharmaceutical Biotechnology and Bioinformatics, Department of Genetic Engineering and Biotechnology, Jashore University of Science and Technology, Jashore 7408, Bangladesh; 14 Department of Pathology, College of Korean Medicine, Kyung Hee University, Seoul 02447, Korea; 15 Korean Medicine-Based Drug Repositioning Cancer Research Center, College of Korean Medicine, Kyung Hee University, Seoul 02447, Korea

**Keywords:** thymidylate synthase, colorectal cancer, molecular docking, ADME, DFT, molecular dynamics simulation, HOMO-LUMO, drug design

## Abstract

Colorectal cancer (CRC) is the second most common cause of death worldwide, affecting approximately 1.9 million individuals in 2020. Therapeutics of the disease are not yet available and discovering a novel anticancer drug candidate against the disease is an urgent need. Thymidylate synthase (TS) is an important enzyme and prime precursor for DNA biosynthesis that catalyzes the methylation of deoxyuridine monophosphate (dUMP) to deoxythymidine monophosphate (dTMP) that has emerged as a novel drug target against the disease. Elevated expression of TS in proliferating cells promotes oncogenesis as well as CRC. Therefore, this study aimed to identify potential natural anticancer agents that can inhibit the activity of the TS protein, subsequently blocking the progression of colorectal cancer. Initially, molecular docking was implied on 63 natural compounds identified from *Catharanthus roseus* and *Avicennia marina* to evaluate their binding affinity to the desired protein. Subsequently, molecular dynamics (MD) simulation, ADME (Absorption, Distribution, Metabolism, and Excretion), toxicity, and quantum chemical-based DFT (density-functional theory) approaches were applied to evaluate the efficacy of the selected compounds. Molecular docking analysis initially identified four compounds (PubChem CID: 5281349, CID: 102004710, CID: 11969465, CID: 198912) that have better binding affinity to the target protein. The ADME and toxicity properties indicated good pharmacokinetics (PK) and toxicity ability of the selected compounds. Additionally, the quantum chemical calculation of the selected molecules found low chemical reactivity indicating the bioactivity of the drug candidate. The global descriptor and HOMO-LUMO energy gap values indicated a satisfactory and remarkable profile of the selected molecules. Furthermore, MD simulations of the compounds identified better binding stability of the compounds to the desired protein. To sum up, the phytoconstituents from two plants showed better anticancer activity against TS protein that can be further developed as an anti-CRC drug.

## 1. Introduction

Colorectal cancer (CRC) is the second deadliest malignancy that has induces an estimated 1.9 million cases and 0.9 million deaths worldwide in 2020. [1]. The developed world accounts for over 63% of all cases related to CRC. However, the rates of CRC have been reported close to 20% in developing countries [2]. Survival of the disease is highly dependent upon the stage of disease diagnosis. It has been revealed that CRC might have a 90% survival rate if diagnosed early stage of the disease, while the rate seldomly touches 10% during stage four [3]. To date, no specific drug treatments of the disease are available. The advancements made in understanding the pathophysiology of the disease have led to increased treatment options including surgery, chemotherapy, and adjuvant radiotherapy to cure the disease [4]. However, the most significant drawback of the treatment is chemotherapeutical resistance. For instance, 5-fluorouracil (5-FU) which is a potent inhibitor of TS enzyme manifests 50% resistance in CRC patients. Although 5-FU-based therapy has been used since the 1950s, the compounds developed resistance against the disease due to overexpression of RAC3 (receptor-associated-activator 3) [5]. The prevalence of CRC has been dramatically growing at an alarming rate globally in recent years [6]. Therefore, the identification of novel therapeutic candidates is an urgent matter.

TS provides the sole *de novo* source of thymidylate for DNA synthesis that helps cell proliferation [7]. During the one-carbon folate metabolic pathway, TS utilizes the dUMP as a de novo source to synthesize the dTMP [8]. The pathway driving TS also catalyzes the transfer of the methyl group as one carbon moiety to dTMP. Additionally, thymidine kinase initiates dTTP (Deoxythymidine triphosphate) formation which is an essential integrated nucleotide in DNA molecules [9]. The enzyme acts as a bottleneck enzyme to induce DNA replication [10], and patients with lower TS expression have a better survival rate in case of CRC-related disease [11]. Hence, inhibition of the enzymes can be utilized as a better treatment option against the disease. 

Natural products originate as secondary metabolites from different sources including bacteria, fungi, and plants. They are chemically diverse molecules that act as a remarkable class of therapeutics to heal various diseases including cancers [9]. The plant products that possess therapeutic properties or exert a beneficial pharmacological effect on the human or animal body are termed medicinal plants [12]. *C. roseus* (drug-Vinblastine) is a type of medicinal plant in which phytoconstituents showed effective activity against different cancers and microbial diseases [13]. However, the effectiveness of the plant and derived compounds against CRC is remains unknown [14]. Other medicinal plants, namely *A. marina*, have also shown potential anticancer activity as well as effectiveness against other viral diseases [15]. The mangrove species, which originated in South Africa and the coastal area of the Persian Gulf [16], has been reported as a folk medicine for skin diseases, rheumatism, and ulcers. A previous study also revealed that *A. marina*-derived flavonoid and phenol contents exhibit significant anticancer activity in hepatocellular carcinoma and breast cancer [17]. Similarly, *C. roseus* (Apocynaceae family) [18], has been reported to have medicinal properties against diabetes, cancer, hypertension, fever, hemostasis, etc. In addition, more than 100 monoterpenoid indole alkaloids have been identified to have derivatives, vinblastine and vincristine, that have shown anticancer effects [19]. Hence, our study focused on the chemical profiles of these two species to analyze the inhibitory potentiality against the desired protein as well as CRC-related disease.

In silico drug designing has been popularized beyond the approach of the conventional drug discovery process [20]. Natural compound extraction and characterization for anticancer drug development usually encompass some unavoidable barriers and are time-consuming [21]. In contrast, computer-aided drug design (CADD) overcomes this limitation and makes the process easy to screen, identify, and characterize new drug candidates within a short time [22]. For example, the CADD-mediated drug development against lung and prostate cancer has been previously reported [23]. CADD study includes molecular docking and molecular dynamics (MD) simulation approaches that identifies effective treatments of different diseases [24]. Molecular docking analysis helps to initially screen drug candidates to the desired target with the favorable binding ability to draggable ligands [25]. Similarly, MD simulations help us understand the stability of protein-ligand interactions in an artificial environment similar to the human body [26]. Therefore, this study utilized computational drug design approaches to sort out the potential drug candidates from the selected plants as a treatment option against CRC.

## 2. Results

### 2.1. Phytochemical Retrieval and Preparation

The Indian Medicinal Plants, Phytochemistry, And Therapeutics (IMPPAT) compound library was used to find the available compounds of the preferred plant [3], and the PubChem database was used to retrieve the phytochemicals compounds. A total of 63 compounds were identified from *C. roseus* (46) and *A. marina* (17) through the abovementioned databases listed in Table S1. The phytochemicals of the two plants have been searched in PubChem through the smile identity and saved in a 2D (SDF) file format. The compounds were prepared and optimized, then converted into pdbqt file format and saved for further examination.

### 2.2. Active Site Identification and Receptor Grid Generation

An active site (AS) is a position of an enzyme or protein that allows macromolecules to bind with a specific molecular substrate. The AS of a protein is formed by different amino acid (AA) residues known as the binding site of the protein. The binding sites of the protein help make a temporary bond with the substrate. The binding site of a protein or nucleic acid can recognize a ligand and make a good binding interaction with the protein, enabling a chemical substrate to undergo a catalyzed reaction as well as helping to stabilize the reaction intermediates. Therefore, the study initially identified the active site position as well as the residues of the binding site for further experiments.

**Figure 1:**
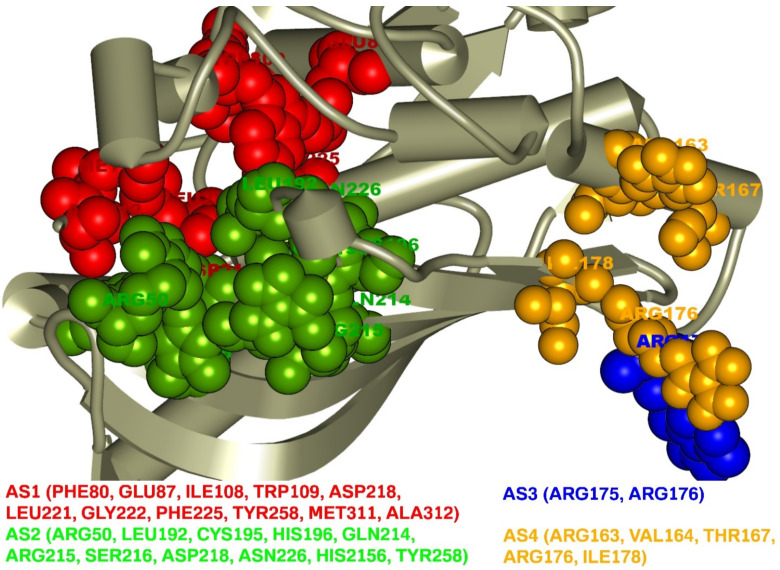
Active site and correspondence binding site of thymidylate synthase. Ball shape with red, green, blue, and yellow representing AS1, AS2, AS3, and AS4, respectively, with their binding site position of the thymidylate synthase.

Analysis of the TS protein identified four AS (AS1, AS2, AS3, AS4) pockets with different binding site residues shown in Figure 1. A total of 27 AS residues corresponding to the four active site pockets was found in this study. The first active site AS1 pocket of the protein was formed with the help of 11 AA residues including PHE80, GLU87, ILE108, TRP109, ASP218, LEU221, GLY222, PHE225, TYR258, MET311, and ALA312. The AS2 pocket that was represented in ball shape in green was also composed of 11 AA residues (ARG50, LEU192, CYS195, HIS196, GLN214, ARG215, SER216, ASP218, ASN226, HIS2156, TYR258) as binding sites of the TS enzyme. The AS3 in blue displayed binding site residue of the TS enzyme with only two residues (ARG175, ARG176), where five residues (ARG163, VAL164, THR167, ARG176, ILE178) formed at the AS4 represented in ball shape with yellow. 

The receptor grid generation is required to specify the active site of the target protein and identify favorable molecules binding to the catalytic sites. Without the receptor grid generation PyRx, it is not able to conduct ligand-based molecular docking with the targeted protein. At the very beginning of the protein preparation process, receptor grid generation requires a ‘prepared’ protein with sufficient bond orders and formal charges that were performed in this study. Grid box generation provides increasingly accurate scoring of ligand poses. Therefore, a receptor grid to the target enzyme was generated based on the previously obtained binding site residues of the protein to achieve more precise scoring of our ligand poses. The grid box with the dimensions X = 57.0465, Y = 45.5471, and Z = 55.7095 in angstrom (Å) was discovered and used for molecular docking simulation.

### 2.3. Molecular Docking Analysis

**Figure 2:**
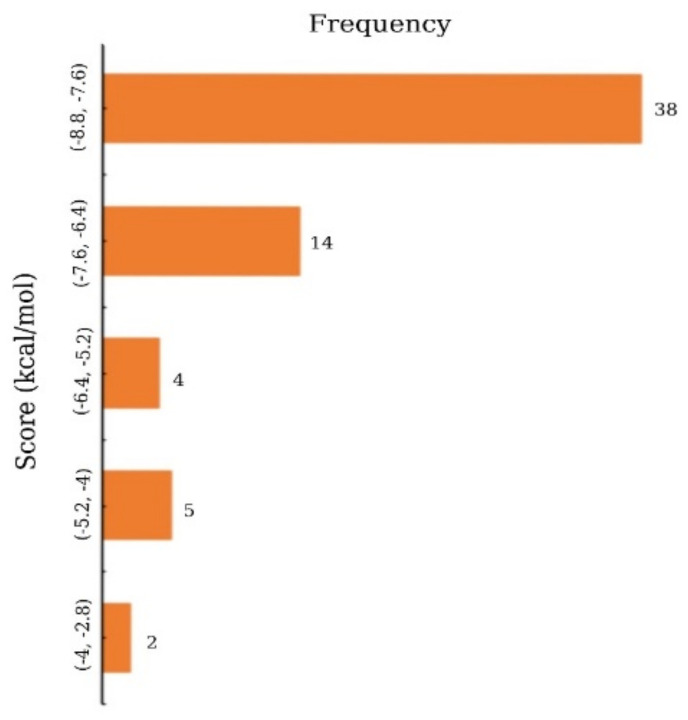
Frequency distribution of 63 phytochemicals over the range of docking score.

Molecular docking is a simulation method for determining how a macromolecule and a drug-like small molecular candidate interact with each other. Primarily, molecular docking analysis was conducted to determine the best intermolecular interaction between the target protein and phytochemical compounds by filtering out those that do not fit into the binding site of the receptor. PyRx tool AutoDock Vina wizard was used to perform the molecular docking of the 63 phytochemical compounds and the protein of interest. The binding affinities found after molecular docking of the phytochemicals compound varied from −8.8 to −3.5 kcal/mole as displayed in Figure 2 and listed in Supplementary Table S1.

Based on the binding affinity and toxicity, the top five percentage (%) of 63 phytochemical (total 4) compounds were selected, which had a better binding affinity compared to the control compound, nolatrexed. In our study, nolatrexed, which showed a binding affinity of −7.7 kcal/mol, was used as a control ligand [27]. Table 1 shows the top four compounds with the highest binding affinity, as well as the binding affinity of nolatrexed.

### 2.4. PK Properties

Pharmacokinetics (PK) is derived from the Greek words *pharmakon* (drug) and *kinetikos* (movement). It is a branch of pharmacology that focuses mainly on the dynamic movements of a small molecular candidate into the body and observes the ADME (absorption, distribution, metabolism, and excretion) properties of a drug-like compound [28]. PK is a type of xenobiotic control process that is required during preclinical studies and the drug development process. It employs a variety of mathematical calculations to provide a model to observe the ADME properties of foreign chemicals (xenobiotics) in the body over time. Analysis of PK properties helps to consider and anticipate biological effective drug-like candidates. The drug-like properties of the four selected compounds were analyzed according to the ‘Lipinski rule of Five’ to develop a lead compound for anticancer activity. The Swiss ADME server was used to analyze the PK properties of the selected compounds, and the ADME properties such as lipophilicity, plasma protein binding, water solubility, and drug-likeness of the compounds were evaluated and listed in Table 2 and Table S2. The PK properties of all the selected compounds were found to be efficient in this study.

### 2.5. Toxicity Prediction

Toxicity prediction is an essential step in the modern drug development process that aids in determining the negative effects of a compound on humans, livestock, plants, and the environment. In silico toxicology is a form of toxicity evaluation that employs numerical methods to study, model, visualize, and forecast chemical toxicity [29]. Traditional drug development methods rely on a variety of animal experiments to assess a compound’s toxicity, which is time-consuming, expensive, and needs ethical considerations [30]. Computer-aided toxicity testing compared to traditional approaches provides a fast and inexpensive method to screen highly toxic chemical compounds that reduces the number of biological experimental tests. Therefore, in a silico toxicity test was conducted using the admetSAR 2.0, proTox-II, and pkCSM web server to determine the negative effects of the four compounds listed in Table 3. 

### 2.6. Interpretations of Protein–Ligands Interaction

**Figure 3:**
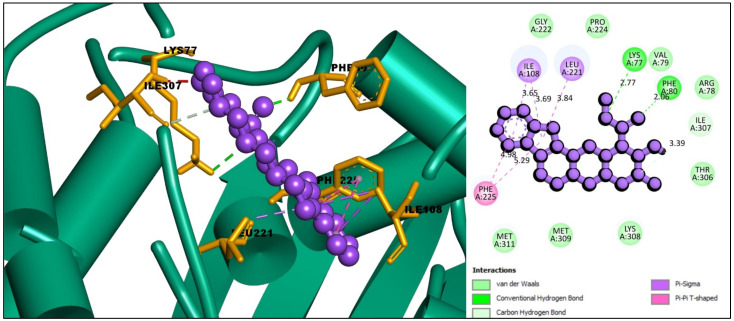
Interaction between the compound CID: 102004710 and thymidylate synthase. The left side represents 3D and the right side represents 2D complex interaction.

The interaction between the selected ligands with the desired protein was observed using the BIOVIA Discovery Studio Visualizer tool. Three Pi-Sigma bonds were found at the positions of ILE108 (3.69 Å), ILE108 (3.65 Å), and LEU221 (3.84 Å), as well as two Pi-Pi T-Shaped bonds at the positions of PHE225 (4.98 Å) and PHE225 (5.29 Å) for the compound CID: 102004710. One conventional hydrogen bond and one carbon-hydrogen bond at PHE80 (2.86 Å) and ILE307 (3.39 Å) position, respectively, were also found to form the compound CID: 102004710 shown in Figure 3 and listed in Table 2. 

**Figure 4:**
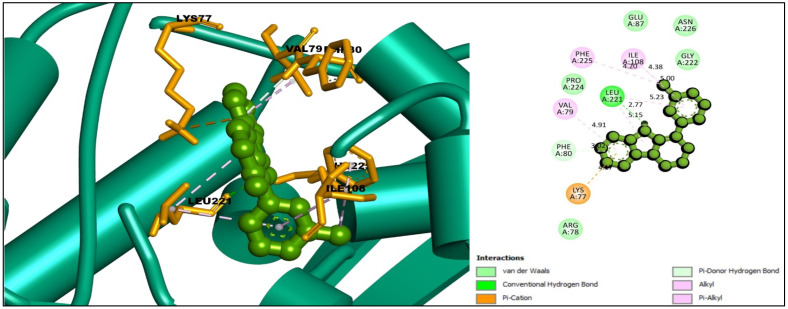
Interaction between the compound CID: 198912 and thymidylate synthase. The left side represents 3D, and the right side represents 2D complex interaction.

For the compound CID: 198912, several Pi-Alkyl bonds were formed with the desired TS enzyme. Five Pi-Alkyl bonds were formed at the positions of PHE225 (4.20 Å), LEU221 (5.15 Å), VAL79 (4.91 Å), ILE108 (5.00 Å), and LEU221 (5.23 Å). One Alkyl bond at ILE108 (4.38 Å) and two Pi-Donor hydrogen bonds were noted at the positions of LYS77 (4.17 Å) and PHE80 (4.04 Å). In addition, at the position of LYS77 another Pi-Cation bond was found, and one conventional hydrogen bond was found at the position LEU221 (2.77 Å) as shown in Figure 4 and Table 2. 

**Figure 5:**
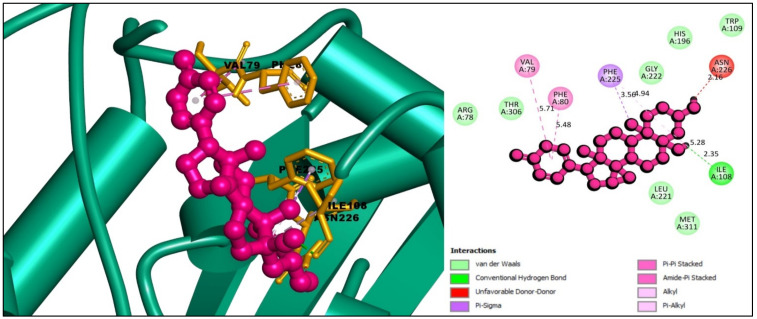
Interaction between the compound CID: 11969465 and thymidylate synthase. The left side represents 3D and the right side represents 2D complex interaction.

The interaction study of the compound CID: 11969465 found one conventional hydrogen bond at the position of ILE108 (2.35 Å) and one Pi-Sigma bond at the PHE225 (3.56 Å) residual position. It also formed one Pi-Pi Stacked and one Amide-Pi Stacked bond at the positions of PHE80 (5.48 Å) and VAL79 (5.71 Å), respectively, where an Alkyl bond formed at ILE108 (5.28 Å) and a Pi-Alkyl interaction formed at PHE225 (4.93 Å) position depicted in Figure 5 and Table 4. 

**Figure 6:**
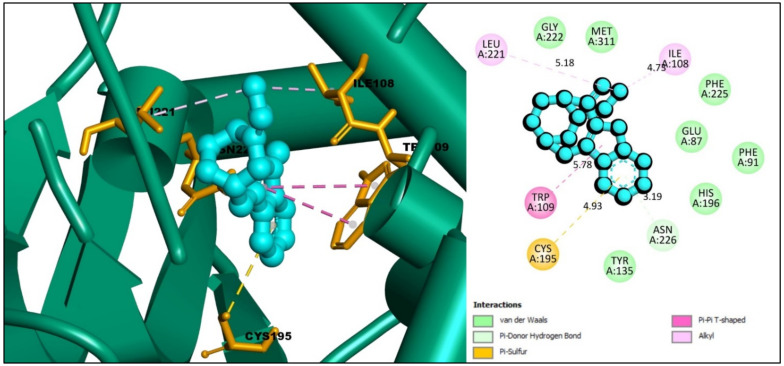
Interaction between the compound CID: 5281349 and thymidylate synthase. The left side represents 3D, and the right side represents 2D complex interaction.

In the case of compound CID: 5281349, the Pi-Donor hydrogen bond was observed only at the ASN226 (4.09 Å) position, where one Pi-Sulfur also has found with CYS195 (4.93 Å). At the position of TRP109, two Pi-Pi T-Shaped bonds were observed with different distances at 5.32 Å and 4.76 Å. It also formed two Alkyl bonds with ILE108 and LEU221, where the distance for the Alkyl bond was 4.73 Å and 5.12 Å, respectively, shown in Figure 6 and Table 4. 

### 2.7. Geometry Optimization and Theoretical Quantum Chemical Calculation

**Figure 7:**
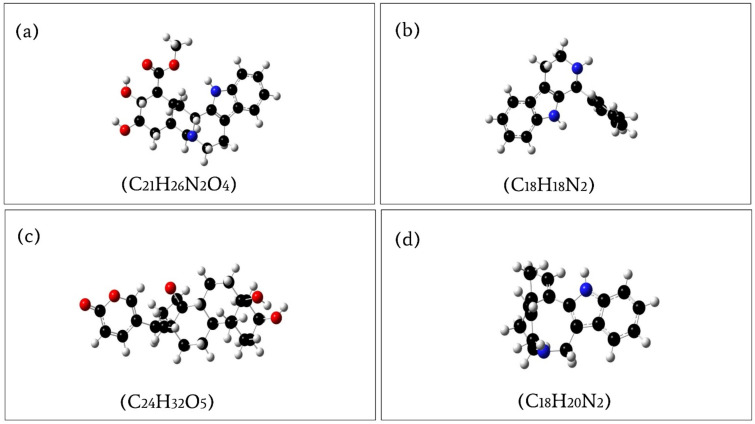
Optimized compounds of (**a**) PubChem CID: 102004710; (**b**) PubChem CID: 198912; (**c**) PubChem CID: 11969465; (**d**) PubChem CID: 5281349, calculated by the B3LYP/6-31G (d, p) density functional theory.

Geometry optimization is a quantum chemical technique used by most chemists to find stable compounds. The methods take rough geometric approximations of a chemical structure and make them as exact as possible. Energy minimization is essential to determining the proper molecular arrangement in space since the retrieved chemical structures are not always energetically favorable. The optimized structure with the lowest energy is the most stable because molecules with the lowest energy state spontaneously decrease their emitted energy. Therefore, the selected compounds were optimized using DFT with Becke’s three-parameter exchange function (B3) with a mixture of HF with DFT exchange terms associated with the gradient corrected correlation function of Lee, Yang, and Parr (LYP) and 6-311G(d, p) basis set [31]. The energy of the selected optimized compounds CID: 102004710, CID: 198912, CID: 11969465, and CID: 5281349 was 1225.948320 a.u, −806.070280 a.u, −1309.527491 a.u, and −807.213195 a.u, respectively, represented in Figure 7 and listed in Table 5. Additionally, the bond angles, bond lengths (bohr, angstroms), and torsional angles optimized during the process are provided in the Supplementary Materials text file format (renamed as Geometry). The dipole moment (μ) measured in Debye, is the electronic parameter resulting from the uneven distribution of charges on different atoms in a molecule. An increase in dipole moment also increases the deformability energy, which will help make the adsorption of the inhibitor easier on the metal surface. Therefore, the dipole moment of the compounds CID: 102004710, CID: 198912, CID: 11969465, and CID: 5281349 was also calculated, which were 3.878234, 2.516531, 9.732138, and 2.127067 Debye (Table 5), respectively, indicating the selected compound should be easier to adsorb on the surface of the metal. 

### 2.8. Frontier Molecular Orbital HOMO/LUMO Calculation

**Figure 8:**
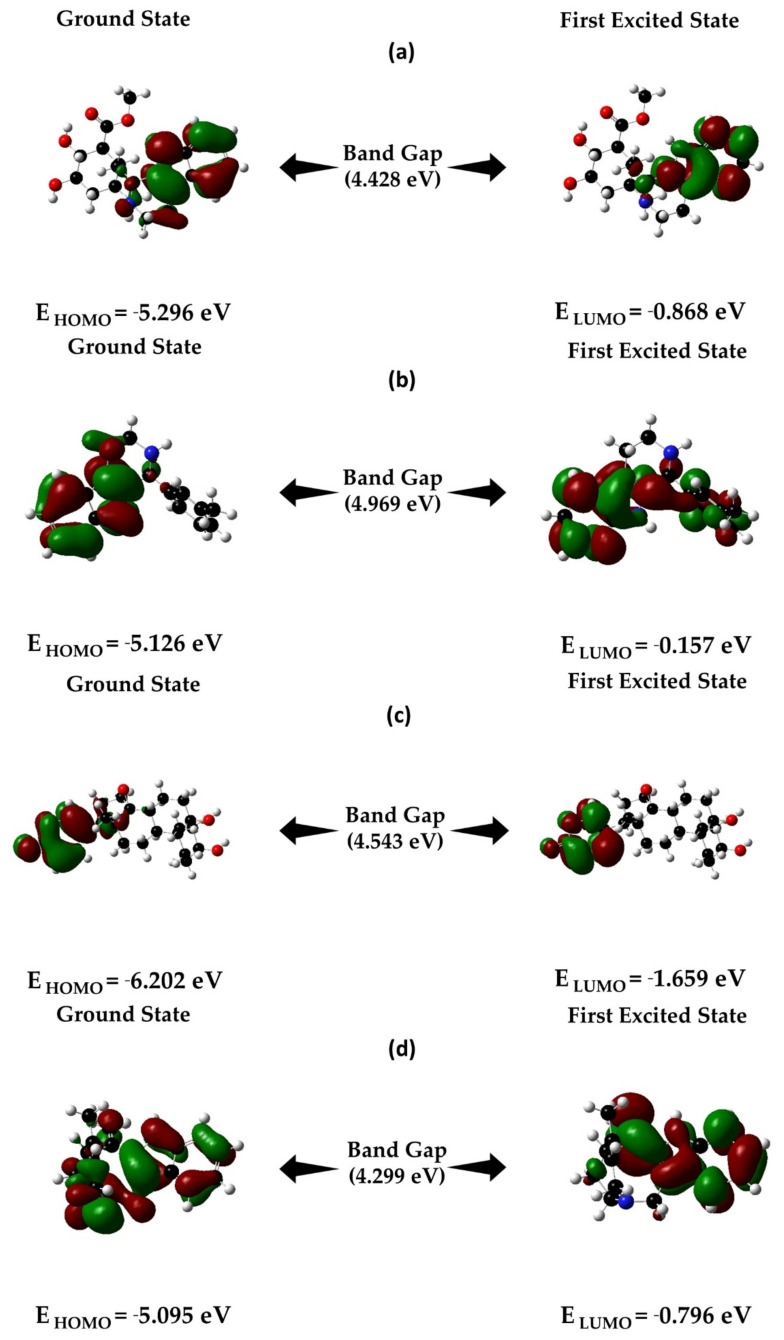
Energy differences and HOMO-LUMO bandgap: (**a**) CID: 102004710; (**b**) CID: 198912; (**c**) CID: 11969465; (**d**) CID: 5281349 obtained by the DFT/B3LYP/6-31G level of theory.

In the field of organic chemistry, frontier molecular orbital (FMO) theory is widely used to illustrate the reactivity and electronic properties of molecules in the transition state to HOMO/LUMO interaction. HOMO stands for highest occupied molecular orbital and determines the capacity of electron-donating to nearest orbitals, whereas the lowest unoccupied molecular orbital (LUMO) indicates the ability of atoms to accept an electron. As a high HOMO-LUMO orbital gap indicates lower chemical reactivity and high kinetic stability, hence we evaluated the HOMO LUMO gap of the selected compounds. The HOMO, LUMO, and HOMO-LUMO, gap energy was calculated by using Gaussian 09 tools and is represented in Figure 8. 

B3LYP/6-31G (d, p) density functional theory was also used in this study to examine the electronic properties of the donor-bridge-acceptor molecular system and determine the quantum chemical properties, including ionization potential (IP), electronic affinity (EA), global hardness (η), global softness (S), chemical potential (µ), electronegativity (χ), and the electrophilicity index (ω) of the four optimized selected molecular structures (Table 6). With the help of the theoretical methods, the ionization potentials (IPs) that help determine the amount of energy required to remove an electron from an isolated atom or molecules such as CID:102004710, CID: 198912, CID: 11969465, and CID: 5281349 were calculated as 5.296 eV, 5.126 eV, 6.202 eV, and 5.095 eV, respectively (Table 6). 

The global hardness and softness and the associated hard/soft acid/base (HSAB) principle were used in this study to observe the reactivity patterns of the molecules and are listed in Table 6. Additionally, the electrophilicity index (*ω*) was calculated to determine the energy required for the system to get saturated and was a range from 1.4 to 3.04 (Table 6), indicating all the selected compounds have high attractive electron power. 

### 2.9. MD Simulations Analysis

MD simulations help determine the stability of protein-ligand complexes in a real-life-like artificial environment. Therefore, to investigate the stability of the phytoconstituents in a complex with the desired protein, a 50 ns MD simulation was performed. To demonstrate the stability of the chosen protein–ligands complex, simulations were run with the complex docking structure and compared with the reference antagonist that binds with TS. The RMSD (root-mean-square deviation), RMSF (root-mean-square-fluctuation), intramolecular hydrogen bonds (Intra HB), and protein-ligand contact analysis (P–L contact) were used to characterize the MD simulation findings.

#### 2.9.1. RMSD Analysis

The RMSD helps characterize and determine the local conformational change of a protein in a complex with the molecules. The RMSD of complex structures is completely acceptable when the average change of the structure remains between 0.1–0.3 nm from a specific time frame to a given time frame. However, a range order that crosses the limit indicates that a large conformational change occurred within the protein structure. The equilibration of the given system relies on the order of the fluctuation rate. An RMSD of the complex system can be calculated by using the Equation (1). 

#### 2.9.2. RMSD of Protein

**Figure 9:**
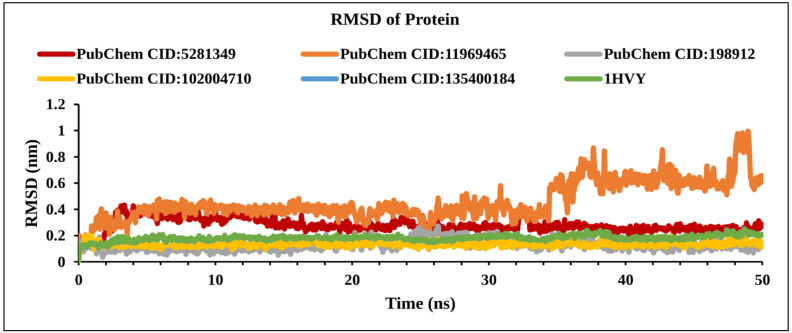
Line graph showing RMSD values of the complex structure extracted from Cα atoms, viz: PubChem CID: 5281349 (dark red); PubChem CID: 198912 (gray); PubChem CID: 135400184 (blue); PubChem CID: 1196465 (orange); PubChem CID: 102004710 (yellow), and 1HVY protein backbone (green) to 50 ns simulation time.

The RMSD of the compounds in complex with TS enzymes was calculated from Cα atoms. An average fluctuation of RMSD in a range between 0.1 nm and 0.3 nm within the reference protein should be considered as stable. However, a much larger fluctuation implies major conformational changes and indicates the system is not stable. The compounds CID: 198912 (gray), CID: 102004710 (yellow), and apo-protein (green) showed stability during the MD simulation with an acceptable RMSD value of less than 0.3 nm (Figure 9). On the other hand, the compound CID: 11969465 showed peak variation from 0.6 nm to 1nm (between 35 ns and 50 ns), indicating that this compound has some rearrangement in the active site compared to the docked conformation. 

#### 2.9.3. RMSD of Ligand

**Figure 10:**
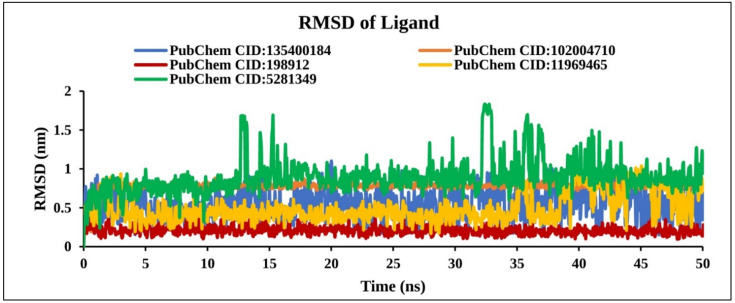
Line graph showing RMSD values of the complex structure extracted from ligand atoms viz: PubChem CID: 135400184 (blue); PubChem CID: 198912 (dark red); PubChem CID: 5281349 (green); PubChem CID: 102004710 (orange); and PubChem CID: 11969465 (yellow) to 50 ns simulation time.

The RMSD of the ligand was calculated from the lig-fit-protein atoms of the complex structure. Analysis of our four compounds RMSD data obtained from the protein fit ligands atom exhibited the lowest fluctuation <1 nm except for CID: 5281349 compounds >1.8 nm (Figure 10). However, two natural compounds (CID: 198912 and CID: 102004710) found stable RMSD at a different position within 0 to 50 ns than the control compound (CID: 135400184). Therefore, it is important to keep in mind that, the molecules can move freely from their original binding site. 

#### 2.9.4. RMSF Analysis

**Figure 11:**
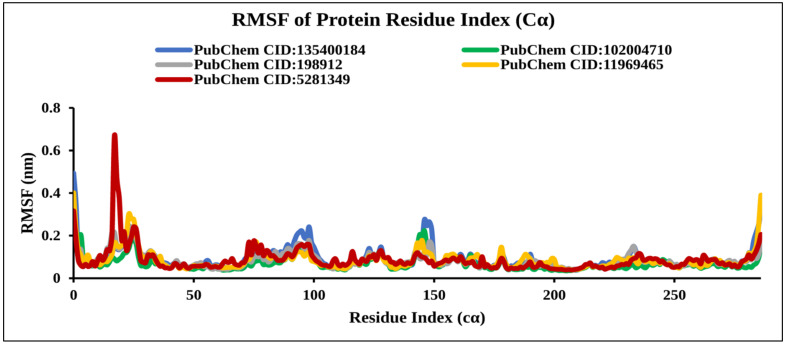
Line graph showing RMSF values of the complex structure extracted from protein residues Cα atoms, viz: PubChem CID: 135400184 (blue); PubChem CID: 198912 (gray); PubChem CID: 5281349 (dark red); PubChem CID: 102004710 (green); and PubChem CID: 11969465 (yellow) to 50 ns simulation time.

The RMSF is important to observe the local changes of a protein that help measure the displacement of a specific atom compared to the reference structure by calculating the average change observed over the number of atoms [32]. The RMSF was calculated by the index of the residue Cα from the complex with TS protein for the selected natural compounds CID: 198912, CID: 5281349, CID: 102004710, CID: 11969465, and the control compound CID: 135400184 as shown in Figure 11. Analysis of the RMSF graph revealed extreme fluctuations were reached between 16 and 26 residues for CID: 5281349 with a maximum range of 0.7 nm, indicating less stability of the compounds. However, the compounds CID: 198912 and CID: 102004710 showed low fluctuations compared to the control compounds, suggesting stability of the system. 

#### 2.9.5. Solvent Accessible Surface Area Analysis

**Figure 12:**
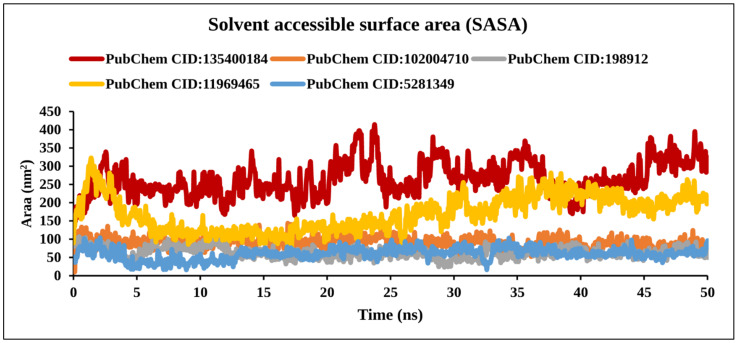
Line graph detailing the solvent-accessible surface area of TS and four compound complexes. The solvent accessible surface area (SASA) of the selected compound is represented by the Y-axis and the time frames are represented by the X-axis.

The SASA calculations were carried out for the TS protein and the TS–Ligand docked complexes and are represented in Figure 12 and Figure S2. 

The SASA values of the TS protein for the AA residues which were involved in bond formation (LYS77, VAL79, PHE80, ILE108, TRP109, CYS195, LEU221, PHE225, ILE307) and spatially nearby residues in the binding site decreased after docking when compared with that before docking. The decrease in SASA values confirmed that these AA residues were involved in the bond formation with the ligand molecules. 

#### 2.9.6. Protein-Ligand Contact Analysis

**Figure 13:**
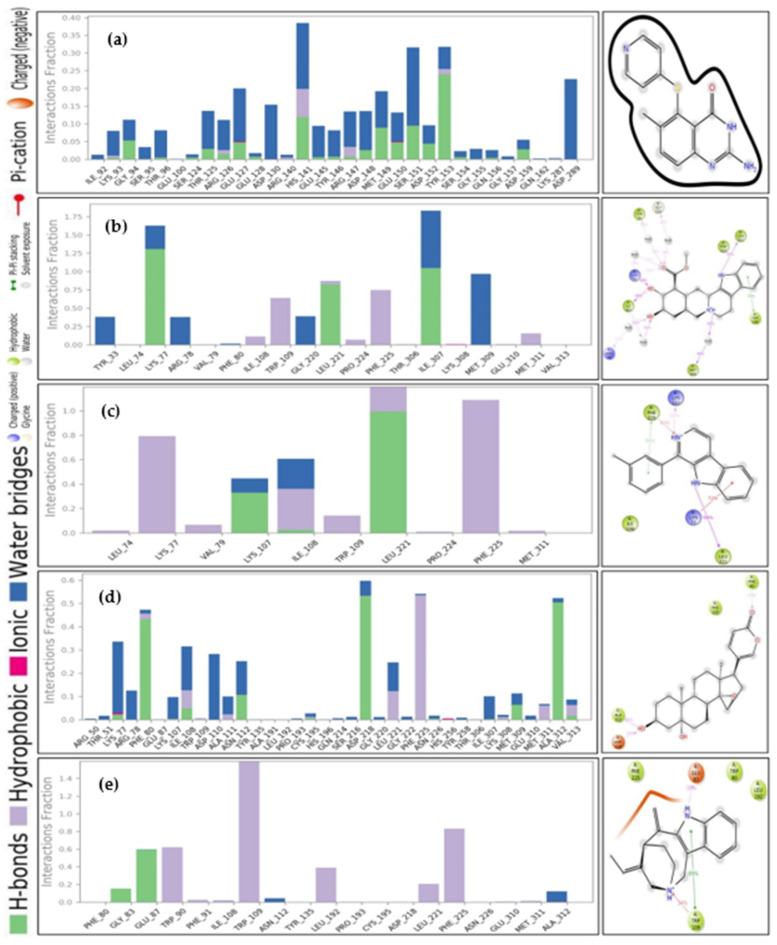
Bar graphs presenting ligand-protein interaction of the: (**A**) PubChem CID: 135400184; (**B**) PubChem CID: 102004710; (**C**) PubChem CID: 198912; (**D**) PubChem CID: 11969465; and (**E**) PubChem CID: 5281349 compounds to 50 ns MD simulation.

The Hydrogen bond, hydrophobic and water bridges bond of protein-ligand interaction play an important role influencing drug specificity, metabolization, and adsorption. Therefore, the intermolecular interaction of the protein-ligand complex was identified via the ‘Simulation Interaction Diagram (SID)’ during the 50 ns simulation. The protein and ligands, including CID: 135400184, CID: 102004710, CID: 198912, CID: 11969465, and CID: 5281349, interaction fraction value was calculated and depicted Figure 13. The compounds CID: 102004710, CID: 198912, and CID: 11969465 showed the highest interaction fraction value of 1.25, 1.0, and 0.5 at LYS 77, LEU 221, and ASP 218 AA residues, respectively formed by multiple bonds. The maximum water bridges and hydrogen bonds found in compounds CID:102004710 and CID: 11969465 indicate more stability of the compounds. In addition, compound CID: 11969465 was found to form multiple ionic, hydrogen, hydrophobic, and water bridge bonding interactions at LYS 77, PHE 80, ILE 108, and ASP 218 AA residues. The number of hydrogen bonds in a system enhances a drug candidate’s metabolic, and adsorption properties. Therefore, the number of hydrogen bonds was observed through a 50 ns simulation during protein-ligand complex interaction as shown in Figures S1 and S2. Interestingly, all the selected natural bioactive compounds showed tremendous hydrogen bonds with protein residues. On the other hand, the torsion properties of the selected compounds were determined and represented in Figure S3.

## 3. Discussion

CRC with high metastasis rates is a major cause of death worldwide [33]. However, no proper medications are available to treat the disease. Lack of the specificity, insufficient concentrations of traditional chemotherapeutics and resistance of chemotherapeutics at the tumor site and their severe adverse effects necessitate developing new treatment strategies such as designing natural drug candidates to improve pharmacological profiles and reduce adverse effects. Toward this end, this study identified natural anticancer drug candidates by targeting the TS enzyme. 

Over the past few decades, CADD has emerged as an important tool for developing new drug molecules in a more cost-efficient way [34]. CADD is also a specialized discipline that uses statistical techniques to model drug-receptor interactions to decide if a given molecule can bind to a target and, if so, with what affinity [35]. Understanding how ligands bind, interact, and suppress a particular protein will lead to the discovery of drug candidates for a specific disease. CADD techniques have made it easier to understand the action and binding mode of a ligand with a particular target molecule and whether the most common binding modes between a ligand and a protein can be predicted using a molecular docking method. However, small molecules can re-rank as drug candidates against a particular disease because MD simulation can identify the mechanism of complex protein-ligand interactions. In addition, the bioactivity of molecules can be assessed through a theoretical quantum chemical calculation such as HOMO-LUMO band energy calculation as well as geometry optimization.

This study investigated 63 natural phytochemicals by targeting the TS enzyme to fight against human CRC. Initially, the molecular docking approach was performed to screen the phytochemical, and the best four compounds were chosen according to their highest binding affinity. The docking process initially found the four best compounds CID: 102004710, CID: 198912, CID: 11969465, CID: 5281349 with binding scores of −8.8, −8.7, −8.7, and −8.6 kcal/mol, respectively. The binding interaction found good hydrogen and hydrophobic bonding between the protein and the ligands. PK properties of the compounds were monitored to identify the metabolite kinetics of small molecular candidates in the human body. A transient and time-course assessment of drug components in the entire blood, tissue, and target organs can also be accessed based on PK properties. A drug’s efficacy is largely dependent on ADME, which is linked to PK properties. The PK parameters must be optimized during the drug design phase so that they can undergo regular clinical trials and be expressed as a promising drug candidate. Under ADME properties that impair a drug molecule’s permeability through the biological membrane, molecular weight, and polar surface topological region (TPSA) were included. The permeability of a drug candidate can be reduced as the molecular weight increases, while the TPSA relative to permeability is increased as the molecular weight decreases. The logarithm of the target molecule’s inorganic and aqueous phase partition coefficient is referred to as LogP in the sense of lipophilicity. The absorption of a drug molecule is influenced by its lipophilicity. Higher LogP is associated with lower absorption and vice versa. The candidate molecule’s solubility is affected by the LogS rating, which is usually selected to be the lowest [36]. This study evaluated the abovementioned PK properties of the selected compounds that found the optimum value of ADME properties of the selected four compounds.

Toxicity is defined as a condition in which a substance shows adverse effect and can harm an organism. Toxicity is reportedly responsible for 20% of late-stage drug discovery failures. Animal trials are used for toxicity testing, which is a complicated, expensive, and time-consuming process. In silico toxicity analysis, which does not require animal trials and is quick and inexpensive, can be used to justify preclinical drug production [37]. Therefore, the toxicity profile of the selected top four phytochemicals was assessed using in silico methods. The noncarcinogenic properties of the compounds were calculated using data from in silico toxicity test servers. Ames experiments were also used to assess the compounds’ possible genotoxicity by assessing the capacity of reverse mutations. The LD50 primarily offers knowledge on the immediate or acute toxicity of compounds shown to be the most effective in the analysis. This study analyzed most of the toxic parameters of the selected compounds that found good toxicity properties of the compound.

To investigate and optimize the geometry of the compounds, a computational DFT-based QM simulation was performed. The FMO-based HOMO-LUMO energy gap was also calculated to evaluate the chemical reactivity of the compounds. The HOMO-LUMO gap energy found for all the compounds was high >4.20 eV, which confirms the low reactivity correspondence to the bioactivity of the compounds. Additionally, the protein-ligands complex structural stability was validated through a 50 ns MD simulation. The binding position in the active points of the four selected compounds was similar, with the RMSD value average less than 1 nm. According to our findings, the four selected screened compounds with the highest energy interacted with a catalytic residue, which is required to inhibit the TS enzyme. The previous study also reported all the potential candidates contain chemo-preventive and chemotherapeutic activity against cancer [38]. We can suggest that the four lead compounds can interfere with the TS enzyme according to the combinatorial docking and molecular dynamics approach; however, these phytochemicals extracted from plants need to be studied further in the lab to determine their effectiveness and inhibitory potential in an in vitro environment.

## 4. Materials and Methods 

### 4.1. Target Preparation

The three-dimensional (3D) experimental tertiary structure of the TS was retrieved from the RCSB protein data bank (PDB) (https://www.rcsb.org/, accessed on 1 September 2021). The targeted protein structure (PDB ID: 1HVY) having a resolution value of 1.9 Å containing 288 AA with 135.83 kDa molecular weight [39], served as a receptor in the docking process. From the beginning to the end, the target was prepared in BIOVIA Discovery Studio Visualizer 2020 software and all the metal ions, cofactors, and water molecules were removed during the structure preparation. AutoDock Tools were employed for the calculation of gasteiger charges and the addition of hydrogen atoms to the protein.

### 4.2. Compound’s Retrieval and Preparation

In this study, the compound’s information was obtained through a comprehensive online database search of various phytochemical composites containing medicinal plants derived from a natural source that is employed for drug design and discovery processes. A manually curated Indian Medicinal Plants, Phytochemistry, And Therapeutics (IMPPAT) database covering >9500 phytochemicals along with >1742 Indian medicinal plants provided cheminformatic techniques to speed up drug discovery of natural bioactive compounds [40]. From the IMPPAT database, a total of 63 phytochemicals of *C. roseus* [38] and *A. marina* [41] which possess anticancer properties respectively were identified and retrieved for virtual screening (Table S1). To conduct further screening procedures, Simplified Molecular Input Line Entry System (SMILES) formats [42] were retrieved from the PubChem database. Several screening processes were applied to the compounds to select the potential candidates [43]. Initially, all compounds were prepared for docking by assigned correct AutoDock 4 atom types, the ‘torsion tree’ was set, nonpolar hydrogens were merged, and aromatic carbons were detected. Nolatrexed (PubChem CID: 135400184), developed as an antifolate anticancer drug was used as a control in this study [44].

### 4.3. Identification of Binding Site and Receptor Grid Generation 

The active sites are located on the surface of an enzyme that interact with other molecules resulting in a chemical reaction of the enzyme [45]. AS helps chemical compounds form enough contact points to generate good binding with desired enzymes, ensuring optimal and favorable catalytic microenvironments. Therefore, the BIOVIA Discovery Studio Visualizer v19.1.0.18287 (BIOVIA) was used to evaluate the AS and corresponding binding site of the protein to achieve the highest binding affinity of the selected compounds [46]. The PyRx virtual screening tool AutoDock Vina was used for receptor grid generation based on the binding site that was discovered from the protein’s complex AS analysis process [47].

### 4.4. Docking Analysis

A molecular docking technique is specially used because it accurately predicts the binding mode of a small molecule to a targeted macromolecule in CADD [48]. The molecular docking analysis was carried out in this study by using the PyRx tools AutoDock Vina with virtual screening to identify the binding mode of the desired protein with selected phytochemicals. PyRx is an open-source virtual screening platform that can screen libraries of compounds against a specific drug target, which is mainly used in CADD approaches. For docking purposes, the default configuration parameters of the PyRx virtual screening tools were used, and the highest binding energy (kcal/mol) with the negative sign was selected for further assessment. Finally, using the BIOVIA Discovery Studio Visualizer v19.1.0.18287 (BIOVIA), the binding relationship of the protein–ligands complex was observed.

### 4.5. PK Properties Prediction

PK properties in CADD are a crucial part of drug discovery, helping to decide on whether the drug candidate should be applied to the biological system or not [49]. PK properties help and describe the integrity and efficacy of compounds in the early stages of drug design. Therefore, the SwissADME server was used in the study to analyze the PK properties of the natural compounds [36]. 

### 4.6. Toxicity Prediction 

During drug development, it is important to assess the adverse effects of chemical compounds before undergoing a clinical trial. Therefore, toxicity evaluation is an essential part of the drug design process. In this study, the toxicity of the compounds was predicted using the admetSAR, ProTox-II, and the pkCSM web server [50]. 

### 4.7. DFT Method Based Geometry Optimization

The Gaussian 09 program (revision D.01) and gauss view 5.0.8 molecular visualization software were used to perform the theoretical quantum chemical calculations with a computer configuration of 2.20 GHz (224 GB RAM, 4 processors) with Windows 10 pro version. Compound optimization was performed by the implementation of conventional functionals Becke’s (B) [51] three parameters with Lee-Yang-Parr (LYP) [52] functionals (B3LYP) with DFT/6-31G (d,p) method. Moreover, the optimized structure was retrieved for further calculation of EHOMO, ELUMO, HOMO-LUMO band gap, electrostatic potential, electrophilicity index, global reactivity (global hardness and global softness), optimized energy and dipole moment parameter of the compounds. The compound’s reactivity, chemical hardness, and softness calculation play an important role in determining the characteristics and polarizability of the electron’s configuration [53]. More distance between the electrons and the nucleus indicates more reactive molecules and a high polarizable candidate, defined as soft ions. In contrast, hard ions candidates have less distance between electrons and the nucleus, less reactivity, and less polarizable species. Therefore, reactivity descriptors such as chemical potential (µ), hardness (η) softness (S), electronegativity (χ), and electrophilicity index (ω) were calculated in this study using Koopmans’ theorem [54]. Chemical reactivity determinators of global hardness (η) and global softness (S) can be defined by the relation η = (IA − EA)/2, and S = 1/η, chemical potential (µ) defined as µ = −χ, the electronegativity (χ) termed as χ = (IA + EA)/2, and the electrophilicity (ω) can be described by the following equation: ω = µ^2^/2η [55,56,57].

### 4.8. Frontier Molecular Orbital HOMO/LUMO Calculation

In the 1950s the frontier molecular orbital theory was developed by Kenichi Fukui to demonstrate the energy difference between two orbitals, HOMO and LUMO. HOMO represents the highest occupied molecular orbitals and has an electron-donating tendency (nucleophilic), and LUMO represents the lowest unoccupied molecular orbitals and has a tendency to accept an electron (electrophilic) [58]. Frontier molecular orbitals (FMOs) are the only way to determine the molecular interaction with different species, molecular reactivity, softness and hardness of compounds, and kinetic stability. Electrons from the HOMO jump to the LUMO during the electrophilic-nucleophilic reaction, resulting in an energy differential between two molecular orbitals, known as the HOMO-LUMO gap. The HOMO-LUMO band gap is crucial in defining the chemical reactivity of the compounds. The following Equation was used to calculate the energy difference between two molecular orbitals.

$$\Delta E=E_{\mathrm{LUMO}}-E_{\mathrm{HOMO}}$$

where ΔE is the HOMO-LUMO gaps, ELUMO is the energy of the lowest unoccupied molecular orbital, and EHOMO indicates the highest occupied molecular orbital energy.

### 4.9. MD Simulations and Trajectory Analysis

Molecular dynamics (MD) simulation was performed to understand the binding stability, fluctuation, conformational changes, and kinetic behavior of desired compounds to the targeted protein. ‘Desmond v3.6 Program’ of Schrodinger (academic version in Linux environment) was utilized to analyze the MD simulation of the target-ligand complex structure by using the OPLS-2005 force field. The system was solvated by using the TIP3P water model. The boundary condition selected in this study was orthorhombic (box shape), and a buffer box was chosen as a calculation method with a box distance of 10 Å. Charges were electrically neutralized by adding suitable ions such as Na_+_ and Cl_−_ with the concentration of salt 0.15 M. The MD simulations were performed at 300 K and one atmospheric pressure (1.01325 bar) with a 10 ps time interval. The SID module in the Schrodinger package was implied to analyze the quality of the MD simulation and the simulation event. From the simulation trajectories, the root-mean-square deviation (RMSD), root-mean-square fluctuation (RMSF), hydrogen bonding interaction, solvent accessible surface area (SASA), and protein-ligand interaction were evaluated.

#### 4.9.1. RSMD Analysis

RMSD is a standard numerical measurement of average conformational changes between the backbone of a protein structure (target) and the ligand (reference) over a certain time frame compared to a reference time [59]. During the MD simulation, the protein frames and the reference frame backbone were first aligned, and then the system’s RMSD was determined based on the atom selection. RMSD can be calculated from the following Equation (1) with the time frame *x* to the MD simulation during 50 ns.

(1) $${\mathrm{RMSD}}_{X}=\sqrt{\frac{1}{N}}\overset{N}{\underset{i=1}{\sum }}\left({r^{\prime }}_{i}\left(t_{x}\right)\right)-r_{i}{\left(\left(t_{ref}\right)\right)}^{2}.$$

Here, the selected number of atoms is termed as *N*; the reference time is defined by *tref*. After superimposing the frame *x* on the reference frame, *r*′ defines the location of the picked atoms within it; and recording intervals are defined by *t_x_*.

#### 4.9.2. RMSF Analysis

RMSF is presumably like RMSD, but it calculates individual residue flexibility, or how much a given residue flexes during a simulation, rather than reflecting positional differences across complete structures over time. Knowing the number of AA residues *i* for a protein during the MD simulation, the RMSF can be calculated by the following Equation (2) through 50 ns simulation time.

(2) $${\mathrm{RMSF}}_{i}=\sqrt{\frac{1}{T}}\overset{T}{\underset{i=1}{\sum }}<\left({r^{\prime }}_{i}\left(t\right)\right)-r_{i}{\left(\left(t_{ref}\right)\right)}^{2}>$$

Here, the trajectory time is expressed as *T*; the given or reference time is defined as *t_ref_*, and *r*′ represents the position of the selected atoms in the frame *i* after superimposing the reference frame, and (< >) represents the average of the square distance taken over residue b. 

#### 4.9.3. Solvent Accessible Surface Area Analysis

In a protein, solvent accessibility of AAs has an important implication. A protein’s surface area that is in touch with the solvent is known as its solvent accessible surface area. It is based on the three-dimensional structure of the protein molecule and is estimated for the natural form. Protein interactions are frequently followed by major conformational changes. We looked at the links between protein structures and the changes in their conformation that go through when they bind.

## 5. Conclusions

Over the recent decades, many advancements in the treatment of colorectal cancer have been made. However, there is still space for development and improvement in the treatment of CRC. This research study nourishes the applicability of anticancer drug compounds that would be able to trigger pharmacists and medicinal chemists to synthesize new potent and selective drug candidates soon. Recently, TS inhibitors have emerged as a possible therapeutic target to CRC. However, much attention is required to develop more powerful inhibitors of the TS protein. In this study a computational drug design approach was applied to identify inhibitors of the TS protein to hinder the activity of CRC. The study utilized a CADD approach, including molecular docking, ADMET analysis, MD simulation, and QM methods that found four compounds namely 18-Beta-hydroxy-3-epi-alpha-yohimbine, 1-(3-Methylphenyl)-2,3,4,9-tetrahydro-1H-β-carboline, Marinobufagenin, and Apparicine as potential anti-CRC candidates. Although the study is based on computational techniques, further evaluation through different lab-based experiment techniques can help determine the activity of the compound that will provide alternatives for CRC therapy. 

## Figures and Tables

**Table 1:** List of compound identity, chemical name, and two-dimensional (2D) structure of selected best four ligands and nolatrexed (control).

No	Compound ID	Chemical Formula	2D Structure	Score (Kcal/mol)
1	CID:102004710	18-Beta-hydroxy-3-epi-alpha-yohimbine	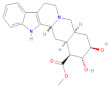	−8.8
2	CID:198912	1-(3-Methylphenyl)-2,3,4,9-tetrahydro-1H-β-carboline	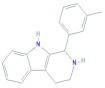	−8.7
3	CID:11969465	Marinobufagenin	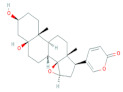	−8.7
4	CID:5281349	Apparicine	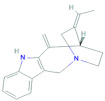	−8.6
5	PubChem CID:135400184	Nolatrexed	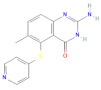	−7.4

**Table 2:** List of pharmacokinetics properties includes physicochemical properties, lipophilicity, plasma protein binding, water-solubility, drug-likeness, and medicinal chemistry of the four selected compounds: medi. chemistry = medicinal chemistry; TPSA = topological polar surface area.

Properties		CID:102004710	CID:198912	CID:11969465	CID:5281349
Physicochemical properties	MW (g/mol) < 500	370.44	262.35	400.51	264.36
	Heavy atoms	27	20	29	20
	Arom. heavy atoms	9	15	26	9
	Rotatable bonds	2	1	1	0
	H-bond acceptors < 10	5	1	5	1
	H-bond donors < 5	3	2	2	1
	TPSA ≤ 140 (A^2^)	85.79	27.82	83.2	19.03
Lipophilicity	Log P_o/w_ ≤ 5	1.71	3.40	3.14	3.30
Plasma protein binding	100%	100%	100%	100%	100%
Water solubility	Log S (ESOL)	−3.49	−4.19	−3.99	−3.54
Pharmacokinetics	GI absorption	High	High	High	High
Drug-likeness	Lipinski	Yes	Yes	Yes	Yes
Medi. Chemistry	Synth. accessibility	Easy	Easy	Easy	Easy

**Table 3:** Drug-induced AMES toxicity, hERG (I) inhibition, carcinogens, rat acute toxicity (LD50 in mol/kg), tetrahymena pyriformis (TP) toxicity, and skin sensitization activity of the four selected compounds. (NC indicates non-carcinogenic).

Parameters	Compounds			
	CID: 102004710	CID: 198912	CID: 11969465	CID: 5281349
Ames toxicity	No	Yes	No	Yes
hERG I inhibition	No	No	No	No
Carcinogens	NC	NC	NC	NC
Rat acute toxicity	2.853	2.82	2.665	2.973
TP toxicity	0.316	0.433	0.352	0.888
HB toxicity	No	Yes	Yes	No
Fish toxicity	No	No	Yes	Yes
Skin sensitization	No	No	No	Yes

**Table 4:** List of binding interactions between four selected phytochemicals with thymidylate synthase.

ID	Residues	Distance (Å)	Bond Category	Bond Type
CID:102004710	PHE80	2.86	Hydrogen Bond	Conventional Hydrogen Bond
	ILE307	3.39	Hydrogen Bond	Carbon Hydrogen Bond
	ILE108	3.69	Hydrophobic	Pi-Sigma
	ILE108	3.65	Hydrophobic	Pi-Sigma
	LEU221	3.84	Hydrophobic	Pi-Sigma
	PHE225	4.98	Hydrophobic	Pi-Pi T-shaped
	PHE225	5.29	Hydrophobic	Pi-Pi T-shaped
CID:198912	LEU221	2.77	Hydrogen Bond	Conventional Hydrogen Bond
	LYS77	4.17	Hydrogen Bond	Pi-Cation
	PHE80	4.04	Hydrogen Bond	Pi-Donor Hydrogen Bond
	ILE108	4.38	Hydrophobic	Alkyl
	PHE225	4.20	Hydrophobic	Pi-Alkyl
	LEU221	5.15	Hydrophobic	Pi-Alkyl
	VAL79	4.91	Hydrophobic	Pi-Alkyl
	ILE108	5.00	Hydrophobic	Pi-Alkyl
	LEU221	5.23	Hydrophobic	Pi-Alkyl
CID:11969465	ILE108	2.35	Hydrogen Bond	Conventional Hydrogen Bond
	PHE225	3.56	Hydrophobic	Pi-Sigma
	PHE80	5.48	Hydrophobic	Pi-Pi Stacked
	VAL79, PHE80	5.71	Hydrophobic	Amide-Pi Stacked
	ILE108	5.28	Hydrophobic	Alkyl
	PHE225	4.94	Hydrophobic	Pi-Alkyl
CID:5281349	ASN226	4.09	Hydrogen Bond	Pi-Donor Hydrogen Bond
	CYS195	4.93	Other	Pi-Sulfur
	TRP109	5.32	Hydrophobic	Pi-Pi T-shaped
	TRP109	4.76	Hydrophobic	Pi-Pi T-shaped
	ILE108	4.73	Hydrophobic	Alkyl
	LEU221	5.18	Hydrophobic	Alkyl

**Table 5:** Molecular structure energies and dipole moments of optimized molecules obtained through DFT calculations.

PubChem CID	Energy (a.u)	Dipole Moment (Debye)
102004710	−1225.94832	3.878234
198912	−806.07028	2.516531
11969465	−1309.527491	9.732138
5281349	−807.213195	2.127067

**Table 6:** Estimated ionization potential (IP), electronic affinity (EA), global hardness (η), global softness (S), chemical potential (µ), electronegativity (χ), and the electrophilicity index (ω) energy of the four optimized selected molecular structures.

PubChem CID	IP (eV)	EA (eV)	η	S	µ	χ	ω
102004710	5.296	0.868	2.214	0.452	−3.082	3.082	2.145
198912	5.126	0.157	2.485	0.402	−2.642	2.642	1.404
11969465	6.202	1.659	2.272	0.44	−3.931	3.931	3.401
5281349	5.095	0.796	2.149	0.465	−2.946	2.946	2.019

## Data Availability

Not applicable.

## Supplementary Materials

The following supporting information can be downloaded at: https://www.mdpi.com/article/10.3390/molecules27072089/s1, Figure S1: Contact mapping of the protein-ligands interactions for the selected four compounds found during the 50 ns simulation run; Figure S2: RMSD (Å), rGyr (Å), intra-HB, MolSA (Å^2^), SASA (Å^2^), and PSA (Å^2^) of the selected four compounds in complex with TS protein; Figure S3: Torsion properties of the selected three compounds; Table S1: List of 63 compounds binding affinity (kcal/mol) with desire protein. Table S2: ADME and PK properties of four selected compounds.

## Sample Availability

There is no sample available.
