# Supplementary material for: Computational Identification of Druggable Bioactive Compounds from Catharanthus roseus and Avicennia marina against Colorectal Cancer by Targeting Thymidylate Synthase
Source: Molecules. 2022 Mar 24;27(7):2089. doi: 10.3390/molecules27072089 (PMC9000506; doi:10.3390/molecules27072089)
Supplement: Supplementary file 1 [file molecules-27-02089-s001.zip › Supplementary Figure.pdf]

Computational Identification of Druggable Bioactive Compounds from *Catharanthus roseus* and *Avicennia marina* against Colorectal Cancer by Targeting Thymidylate Synthase

Md Rashedul Islam [1,2,3], Md Abdul Awal [4], Ahmed Khames [5], Mohammad A. S. Abourehab [6,7], Abdus Samad [8,9], Walid M. I. Hassan [1], Rahat Alam [8,9], Osman I. Osman [1], Suza Mohammad Nur [4], Mohammad Habibur Rahman Molla [10], Abdulrasheed O. Abdulrahman [4,11], Sultana Rajia [3,12], Foysal Ahammad [9,10], Md Nazmul Hasan [8,13,*], Ishtiaq Qadri [10,*] and Bonglee Kim [14,15,*]

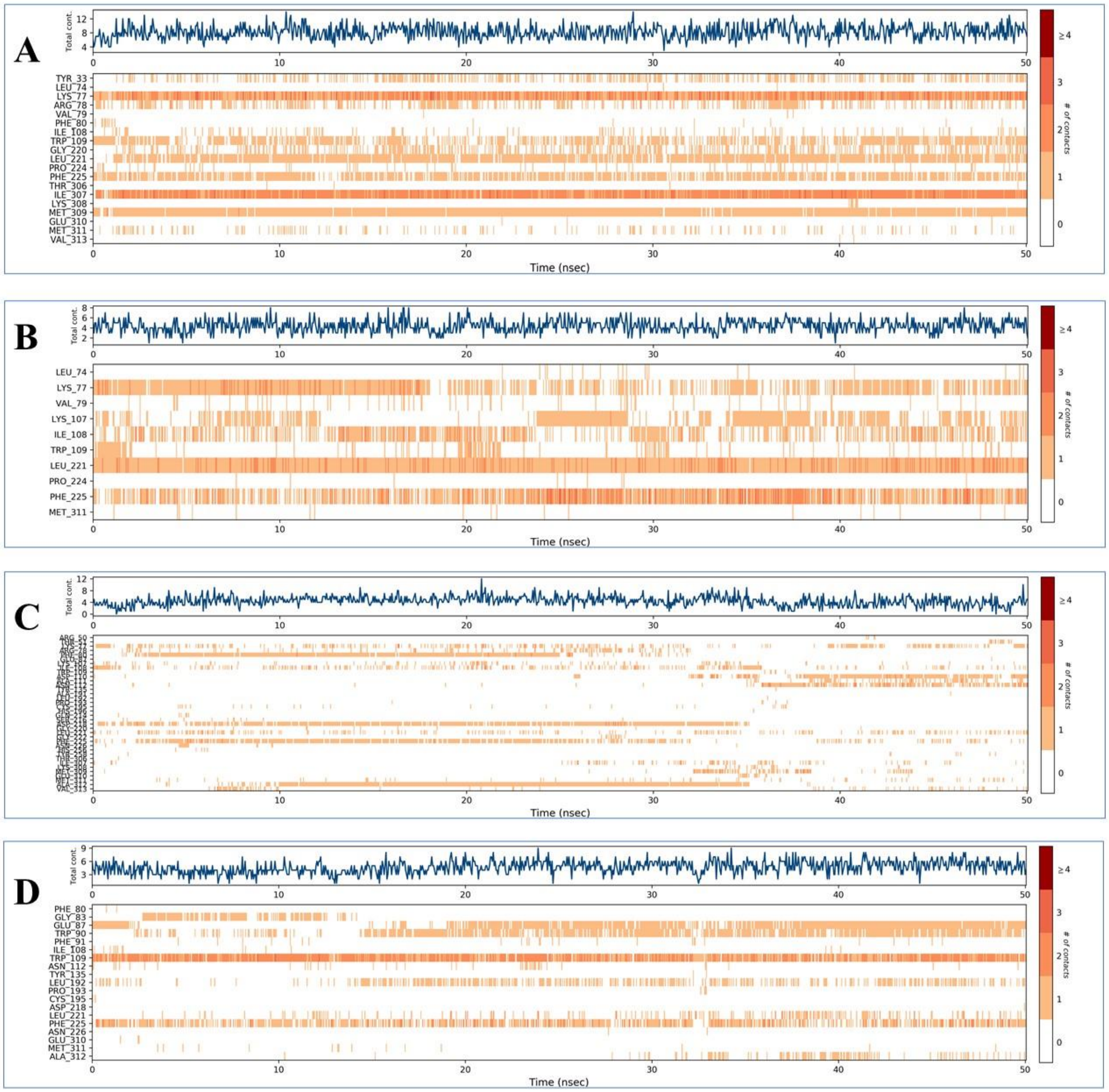

Figure S1: Showing the contact mapping of the protein-ligands interactions for the selected four compounds found during the 50 ns simulation run. Herein, showing the selected four ligands A. PubChem CID: 102004710, B. PubChem CID: 198912, C. PubChem CID: 11969465, and D. PubChem CID: 5281349 contact map with target protein.

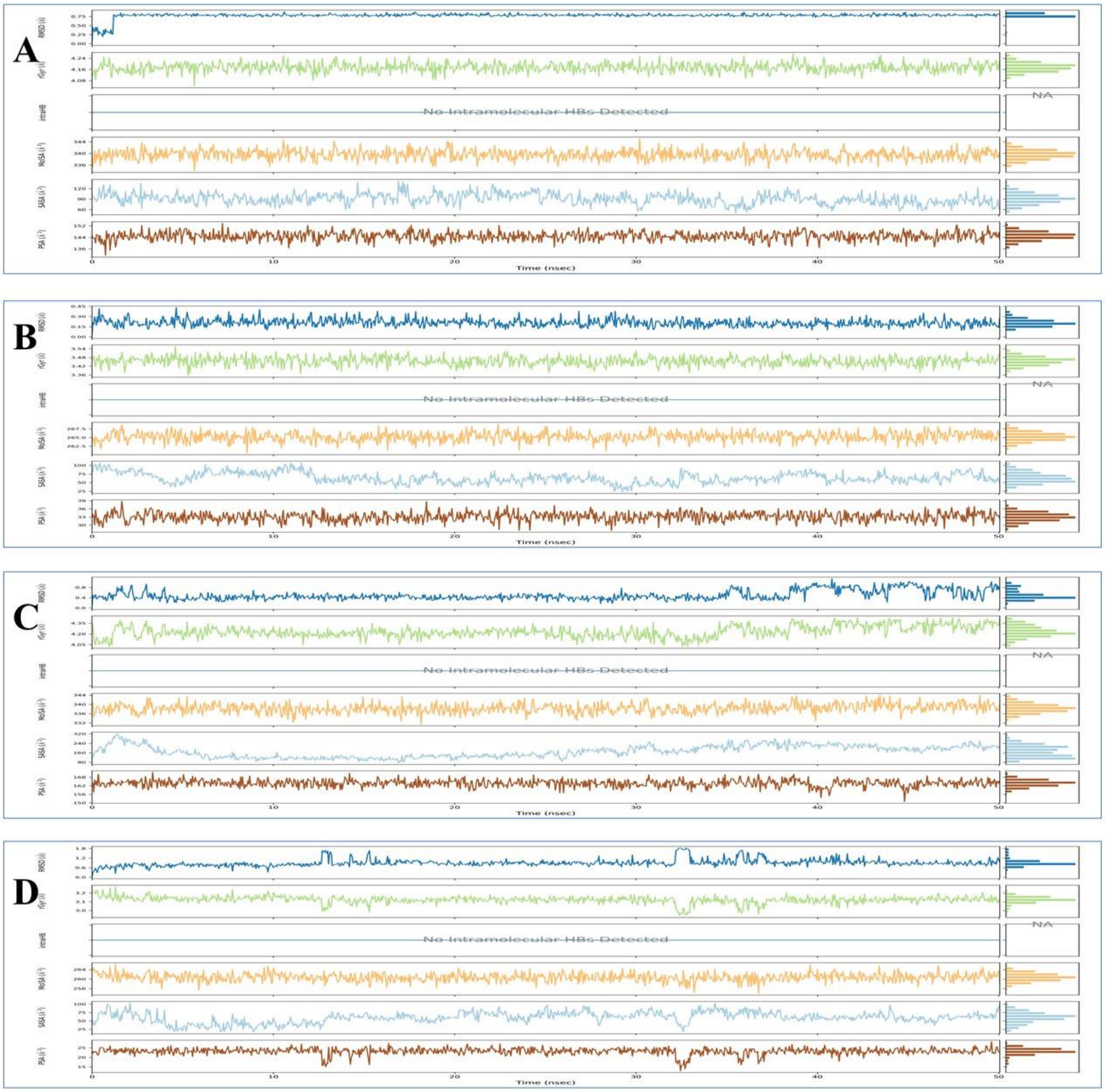

Figure S2: Depicted the RMSD (Å), rGyr (Å), intra-HB, MolSA($Å^2$), SASA($Å^2$), and PSA ($Å^2$) of the selected four compounds in complex with TS protein. Herein, showing the value of the compounds A. PubChem CID: 102004710, B. PubChem CID: 198912, C. PubChem CID: 11969465, and D. PubChem CID: 5281349.

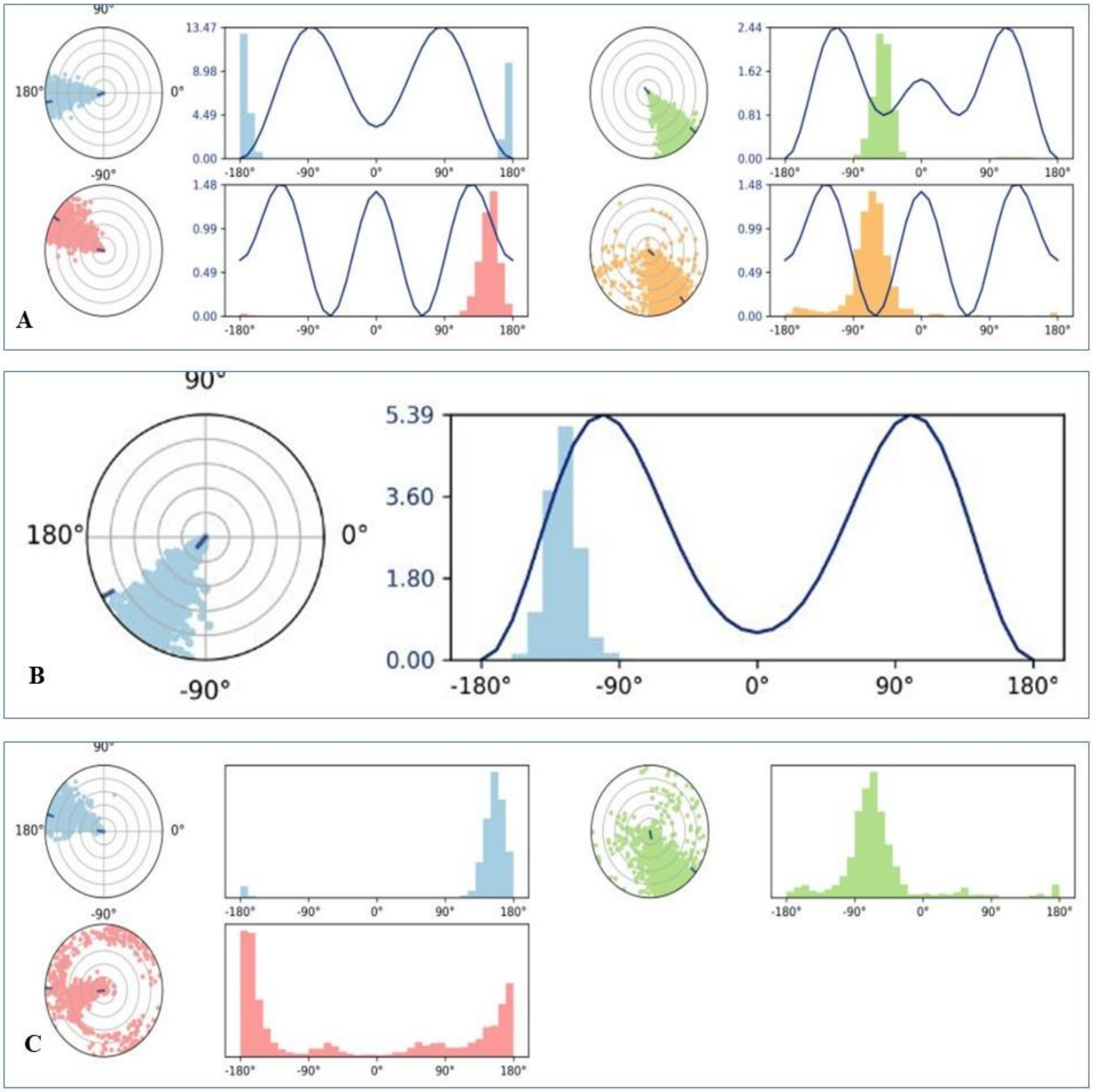

Figure S3: Depicted the torsion properties of the selected three compounds A. PubChem CID: 102004710, B. PubChem CID: 198912, C. PubChem CID: 11969465.
