# Supplementary material for: Computational Identification of Druggable Bioactive Compounds from Catharanthus roseus and Avicennia marina against Colorectal Cancer by Targeting Thymidylate Synthase
Source: Molecules. 2022 Mar 24;27(7):2089. doi: 10.3390/molecules27072089 (PMC9000506; doi:10.3390/molecules27072089)
Supplement: Supplementary file 1 [file molecules-27-02089-s001.zip › Supplementary Table.pdf]

Supplementary Table on

Computational Identification of Druggable Bioactive Compounds from *Catharanthus roseus* and *Avicennia marina* against Colorectal Cancer by Targeting Thymidylate Synthase

Md Rashedul Islam [1,2,3], Md Abdul Awal [4], Ahmed Khames [5], Mohammad A. S. Abourehab [6,7], Abdus Samad [8,9], Walid M. I. Hassan [1], Rahat Alam [8,9], Osman I. Osman [1], Suza Mohammad Nur [4], Mohammad Habibur Rahman Molla [10], Abdulrasheed O. Abdulrahman [4,11], Sultana Rajia [3,12], Foysal Ahammad [9,10], Md Nazmul Hasan [8,13,*], Ishtiaq Qadri [10,*] and Bonglee Kim [14,15,*]

Table S1: A list of 63 compounds binding affinity (kcal/mol) with desire protein.

Compound Name	Binding Affinity
PubChem CID 71315502	-17
PubChem CID 28285721	-10.7
PubChem CID 1008512-18-2	-10
PubChem CID 225689	-9
PubChem CID 102004710	-8.8
PubChem CID 12303662	-8.8
PubChem CID 225688	-8.8
PubChem CID 95695-83-6	-8.8
PubChem CID 198912	-8.7
PubChem CID 11969465	-8.7
PubChem CID 157688	-8.6
PubChem CID 5281349	-8.6
PubChem CID 5316334	-8.6
PubChem CID 100933831	-8.5
PubChem CID 100933832	-8.5
PubChem CID 25109979	-8.5
CHEBI CID 69052	-8.5
PubChem CID 11646359	-8.4
PubChem CID 16219576	-8.4
CHEBI CID 69262	-8.4
CHEBI CID 132821	-8.4
PubChem CID 102399433	-8.3
PubChem CID 25109978	-8.3
PubChem CID C00024521	-8.3
CHEBI CID 69256	-8.3
CHEBI CID 69257	-8.3
PubChem CID 25109977	-8.2
PubChem CID 104380-51-0	-8.1
PubChem CID 12305664	-8
PubChem CID 197026	-8
ChemSpider CID 28288759	-8
CHEBI CID 69264	-8
PubChem CID 100953813	-7.9
PubChem CID 101686461	-7.9
PubChem CID 100925385	-7.8
PubChem CID 161115	-7.8
PubChem CID C00024497	-7.8
PubChem CID C00047645	-7.8
CHEBI ID 52126	-7.8
ChemSpider CID 21379030	-7.6
PubChem CID 2564-23-0	-7.6
CHEBI CID 69261	-7.6
ChemSpider CID 57619488	-7.5
PubChem CID 59706266	-7.5

PubChem CID 327791	-7.4
PubChem CID C00025979	-7.4
CHEBI CID 69263	-7.4
CHEBI CID 49211	-7.4
PubChem CID 161300	-7.3
CHEBI CID 69259	-7.1
CHEBI CID 69265	-7.1
CHEBI CID 69258	-6.7
CHEBI CID 144380	-6.4
CHEBI CID 69260	-6.1
PubChem CID 107561	-5.7
PubChem CID 5460802	-5.7
PubChem CID 12300199	-5.4
PubChem CID 443158	-5.2
PubChem CID 6427789	-4.9
ChemSpider CID 23254674	-4.5
CHEBI CID 83546	-4.5
PubChem CID 145857	-4.1
PubChem CID 23725066	-3.5

Table S2: ADME and PK properties of four selected compounds.

Compound Name	Molecular weight (g/mol)	Heavy atoms	Aromatic heavy atoms	Rotable bonds	H-bond acceptors	H-bond donors	LogP(o/w)	LogS(ESOL)	GI absorption	Plasma protein binding
CID:102004710	370.44	27	9	2	5	3	1.71	-3.49	high	0.859, 100%
CID:198912	262.35	20	15	1	1	2	3.4	-4.19	high	0.903, 100%
CID:11969465	400.51	29	26	1	5	2	3.14	-3.99	high	1.14, 100%
CID:5281349	264.36	20	9	0	1	1	3.3	-3.54	high	0.918, 100%
